# A cross-sectional study on the influence of COVID-19 pandemic on physical activity patterns among residents in a South Indian City

**DOI:** 10.1186/s43161-022-00092-w

**Published:** 2022-08-24

**Authors:** G. Shankar Ganesh, Anandhi Dakshinamoorthy, M. Tamilalagan, Deepali Shah, Saloni Dokania

**Affiliations:** 1 Composite Regional Centre for Skill Development, Rehabilitation, and Empowerment of Persons with Disabilities, Lucknow, Uttar Pradesh 226017 India; 2 Faculty of Medical and Health Sciences, SRM Institute of Science and Technology, Kattankulathur, Chennai, Tamil Nadu 603203 India

**Keywords:** COVID-19, Exercise, Lockdown, Physical fitness, Physical inactivity, Population surveys and questionnaires

## Abstract

**Background:**

Studies have revealed that the COVID-19 pandemic has increased sedentary behavior and reduced the number of physical activities in public. The present study attempted to assess the changes in physical activity patterns among the residents of a south Indian city at different stages after the COVID-19 outbreak.

The present cross-sectional prospective study was conducted on 372 participants between November 2020 and March 2021. The physical activity patterns before, during, and after the lockdown phase were collected using a custom-built questionnaire, and the current level of physical activity was recorded using the international physical activity questionnaire–short form (IPAQ-SF).

**Results:**

Higher number of respondents reported limiting the intensity of physical activities during and after lockdown [(228/372; 61.29%) and (216/372; 58.06%), respectively]. Additionally, respondents reporting lower physical activity intensity [mean total metabolic equivalents of task (MET)/week: 1182.80] compared with (99/372; 26.61%), and (63/372; 16.93%) numbers of participants who engaged in moderate (mean total MET/week-3005.86) and high levels (mean total MET/week-4188.67) of physical activities respectively.

**Conclusions:**

The results of the study reported immediate and long-term impacts on self-reported physical activity patterns among the study sample.

**Supplementary Information:**

The online version contains supplementary material available at 10.1186/s43161-022-00092-w.

## Background

Physical activity can protect the body against obesity, cardiovascular diseases, and type 2 diabetes mellitus; improve cognitive health; and reduce the risk of premature death from any cause [1]. On the other hand, unhealthy dietary habits and physical inactivity can increase the risk of cardiovascular diseases and cognitive decline. Therefore, insufficient physical activity is considered a global public health problem [2]. The Centre for Disease Control and World Health Organization (WHO) recommends 150 min of moderate-intensity physical activity, 75 min of vigorous-intensity physical activity, or an equivalent combination of both per week for people between 18 and 64 years of age [3]. New evidence recommends short (> 10 min) and dispersed physical activity bouts throughout the week [4]. Despite the documented benefits and significance of the physical activity, Indians are more sedentary and lesser physically active than global estimates. Additionally, < fewer than 10% of Indians are engaged in recreational physical activities [5]. Furthermore, the leisure time exercise intensities did not meet the globally recommended intensities [6].

COVID-19 was declared a pandemic by the WHO in March 2020, and a majority of governments worldwide instituted a countrywide lockdown to restrict its spread. The lockdown was extended in five phases in India. During this lockdown, public health orders curtailed the movement outside the home with recommendations to stay at home and practice social distancing. This was compounded by directives to close fitness centers, public parks, and other infrastructure required to engage in a physically active lifestyle. Though high-volume and high-intensity physical activities are not recommended during the pandemic [7], studies have documented the benefits of physical activities on mental and physical health during the lockdown period [8, 9].

Studies have reported an increase in sedentary behavior and a reduction in the number of physical activities undertaken by the public [10, 11] during the COVID-19 pandemic. These findings are similar to the significant decrease in physical activity patterns observed in children and adolescents following the 2011 Japan earthquake and tsunami [12]. Data indicates that physical inactivity and sedentary behavior will persist after the COVID-19 pandemic [13] and become the new societal norm.

No study has been conducted in India to demonstrate the difference in physical activity patterns due to COVID-19 and how people are currently doing physical activity. Therefore, the physical activity pattern during the lockdown and before-lockdown phase must be evaluated to determine the lasting influence of the COVID-19 pandemic. Identification of sociodemographic factors that influence public engagement in physical activities in the Indian environment may assist the government and policymakers develop physical activity guidelines during and after the pandemic in the future. Thus, the present study attempted to document the immediate and late physical activity pattern changes during and after the lockdown phase of the COVID-19 pandemic and investigate the role of selected sociodemographic factors associated with physical activity in an urban setting in the city of Chennai, India.

## Methods

The present community based online survey was conducted in 372 adults of both sexes between 18 and 59 years between November 2020 and March 2021 after institutional ethics clearance. The study and protocol were performed according to the Declaration of Helsinki guidelines and used a self-administered questionnaire distributed through social media platforms.

The sample size was calculated based on previous estimates of the prevalence of physical inactivity across India [5]. Using the OpenEpi V3.01 software (Atlanta USA) with *P* = 50%, relative precision of 20%, confidence limit of 95%, and attrition of 20%, the sample size was calculated to be 287 [14]. The study participants were recruited from Chennai, the capital city of Tamil Nadu, India.

Adults with at least a bachelor degree of education and free of COVID-19 symptoms or those with a negative report of COVID test from the last 6 months were included in the study. Other inclusion criteria included adults providing verbal informed consent residing in Chennai for at least 6 months, having access to a smartphone with internet access, reading and writing English, and comprehending the questionnaire. Participants with any members within their household testing positive for coronavirus (including those on quarantine and self-isolation); those awaiting results of coronavirus testing; persons with physical and mental disabilities or chronic health conditions; pregnant and post-partum females; and patients recuperating from acute medical illness were excluded from the study.

A structured questionnaire (see Supplementary file 1) was used to obtain data on sociodemographic parameters. The participants were asked to recall information regarding their physical activity patterns before (before March 2020), during (March 2020–June 2020), and after (post unlock 3.0; August 2020) the lockdown period of the COVID-19 pandemic. At the time of administration of the questionnaire, the level of physical activity was recorded using the international physical activity questionnaire (IPAQ) [15]. Each item was included in the questionnaire after receiving consensus from all the authors. The questionnaire was tested on 30 random adult populations from the city of Chennai, India. The original questionnaire in English was used for the study purpose to avoid the practical difficulties in getting the questionnaire and the IPAQ short forms back-translated. As English is spoken widely in Chennai, this did not affect the study objectives and outcomes.

Demographic characteristics such as age, sex, body mass index based on self-reporting of height and weight, marital status (single, married, widowed, or separated), employment (employed or not-employed), and education level (graduate, post-graduate, or doctoral-level) were documented. The socioeconomic status (SES) of the participants was determined using the modified Kuppuswamy’s SES scale [16]. The SES was classified into five groups, namely upper (I), upper-middle (II), lower-middle (III), upper-lower (IV), and lower (V) based on per monthly family income. Then, questions were asked regarding their physical activity patterns (before, during, and after the lockdown) and COVID-19 history.

The physical activity (weekly vigorous and moderate exercise, walking, and sitting time) was documented for the week before completing the survey using the IPAQ-short form (IPAQ-SF) [17]. Although the extended version of the IPAQ is slightly more reliable than the short-form, the long-form is lengthy and less understandable [18]. All instructions while calculating the score adhered and responses to the IPAQ-SF were converted into the metabolic equivalent of task (MET) minutes per week (MET-min/week) as per the scoring protocol [17]. The MET-min/week was calculated by multiplying the MET values (walking = 3.3, moderate activity = 4, vigorous activity = 8) by the minutes of activity and the number of days. The overall MET minutes were summed up to obtain the total MET minutes per week, and the physical activity levels were classified as low, moderate, and high.

A physiotherapist network practicing in the urban and peri-urban area of Chennai were contacted by mobile and social media platforms, including WhatsApp groups and explained the study objectives in detail. Those who consented to assist in data collection were provided with a copy of the anonymous self-reported questionnaire along with the IPAQ-SF questionnaire to gather data from potential participants meeting our inclusion and exclusion criteria through e-mail, social media, and WhatsApp. This ensured the avoidance of virus exposure to potential participants.

### Statistical analysis

SPSS (Statistical Analysis System) version 16.0 (IBM Corp., Armonk, NY) was used for statistical analysis. Estimates were expressed as mean ± standard deviation. One-way analysis of variance (ANOVA) (with post hoc Tukey-HSD procedure) was used to compare means of data between the three groups classified based on IPAQ-SF (low, moderate, and high). Furthermore, correlation coefficients were computed using the Pearson product analysis to understand the association between participant characteristics and changes in physical activity levels as reported by IPAQ-SF. Regression analysis was conducted where appropriate, and a *P* value of < 0.05 was considered statistically significant.

## Results

Out of 685 persons identified, the eligibility criteria were met by 420 persons (61.31%). Reasons for exclusions were over or under-age (*n* = 42), presence of chronic diseases (*n* = 152), COVID-19 affected (self or members of household) (*n* = 44) or having symptoms of COVID-19 (*n* = 23), and refusal to participate (*n* = 4). All potential participants were invited to take part in the questionnaire. Data from 48 respondents were excluded due to non-completion of the questionnaire. The data was collected and analyzed from 372 (200 males and 172 females; mean age 38.18; range 18–59 years) consenting participants.

The general characteristics of the study population are presented in Table 1. The change in the physical activity pattern frequency is presented in Table 2. A total of 138 people were engaged in recreational activities before lockdown (37.09%). This frequency reduced during the lockdown (*n* = 86; 23.11%) and increased after lockdown (*n* = 151; 40.59%). However, the IPAQ-SF scores recorded after the lockdown indicated that 56.4% (*n* = 210) of the respondents engaged in low levels of physical activities, 26.6% (*n* = 99) were moderately active (male: 58.3%), and 16.93% (*n* = 63) were highly active.

**Table 1:** General characteristics of the study population

Variable	Males (200)	Females (172)	Total 372
Age [mean (SD)]	39.19 (12.03) (range 18–59)	38.7 (11.05) (range 18–59)	38.18 (11.47) (range 18–59)
BMI (kg/m^2^)	28.1 (5.6)	26.9 (6.6)	27.4 (7.8)
	< 18.5–8	< 18.5–8	< 18.5–16
	18.5–24.9–87	18.5–24.9–81	18.5–24.9–168
	25.0–29.9–64	25.0–29.9–41	25.0–29.9–105
	> 30–41	> 30–42	> 30–83
SES	Lower–13	Lower–08	Lower–21
	Upper-lower–29 Middle–52	Upper-lower–17 Middle–49	Upper-lower–46 Middle–101
	Upper-middle–49	Upper-middle–68	Upper-middle–117
	Upper class–57	Upper class–30	Upper class–87
Education	Bachelor–144	Bachelor–120	Bachelor–264
	Post-graduate–42	Post-graduate–45	Post-graduate–87
	Doctoral–14	Doctoral–07	Doctoral–21
Marital status	Single–82	Single–68	Single–190
	Married–113	Married–87	Married–160
	Widowed/–5	Widowed/-	Widowed/-
	Separated	Separated–17	Separated–22
Employment	Employed–113	Employed–109	Employed–222
	Unemployed–76	Unemployed–59	Unemployed–135
	Retired–11	Retired–4	Retired–15
If yes,	Working from home–71	Working from home–59	Working from home–130
	Working from office–42	Working from office–50	Working from office–92
Associated medical conditions (*n* = 152)	Diabetes 41		
	Hypertension 38		
	Any known heart diseases 13		
	Arthritis 27		
	Any known respiratory diseases 7		
	High cholesterol–23		
	Others (3 thyroid disorders )		

*BMI* Body mass index, *SES* Socio-economic status

**Table 2:** Physical activity patterns of the participants before, during, and after the introduction of lockdown measures

	Before lockdown	During lockdown	After lockdown
**Engagement in physical activities**	° Nature of daily chores (*n* = 372; 200m;172f) • Light–144 (90m; 54f) • Moderate–176 (94m;82f) • Vigorous–52 (16m; 36f)	• Same as previous intensities–105(59m; 46f) • Less than previous intensities–228(126m; 102f) • More than previous intensities–39 (15m; 24f)	• Same as previous intensities –96 (51m; 45f) • Less than previous intensities–216(121m; 95f) • More than previous intensities–60(28m; 32f)
**Engagement in leisure time physical activities**	° (*n* = 138; 93m; 45f): • Light–41 (10m; 31f) • Moderate–78 (70m; 8) • Vigorous–19 (13m; 6f)	° (*n* = 86; 62m; 24f) • Same as previous intensities–45 (37m; 8f) • Less than previous Intensities–30 (17m; 13f) • More than previous intensities–11 (8m; 3f)	° (*n* = 151; 78m; 73f) • New beginners–30 (11m;19f) • Same as previous intensities–96 (53m; 43f) • Less than previous intensities–21 (12m; 9f) • More than previous intensities–4 (2m; 2f)
**Days/week engagement in leisure time physical activities**	° (*n* = 138; 93m; 45f) • All days–10 (8m; 2f) • Minimum 5 days a week–23 (17m; 4f) • Minimum 3 days a week–42 (24m; 18f) • Less than 3 days–63 (44m; 21f)	° (*n* = 86; 62m; 24f) • All days–6 (4m; 2f) • Minimum 5 days a week–20 (13m; 7f) • Minimum 3 days a week–21 (11m; 10f) • Less than 3 days–39 (34m; 5f)	° (*n* = 151; 78m; 73f) • All days–15 (9m; 6f) • Minimum 5 days a week–28 (17m; 11f) • Minimum 3 days a week–44 (14m; 13f) • Less than 3 days–64 • (38m; 43f)

Table 3 shows the average minutes spent in vigorous, moderate, walking, and sitting activities. The difference in the average minutes spent in vigorous activities [*f* (2) = 56.01; *P* < 0.001], moderate activities [*f* (2) = 18.23; *P* < 0.001], walking activities [*f* (2) = 41.05; *P* < 0.001], and total MET minutes [*f* (2) = 127.759; *P* < 0.001] according to the activity level (low, moderate, or high) was statistically significant. However, no difference was observed in the sitting activities [*f* (2) = 36.3; *P* > 0.05] between groups. Tukey post hoc test revealed significant pairwise differences between groups classified as high and low and high and moderate for vigorous and moderate activities (*P* < 0.05). No difference was observed between the moderate and low groups for vigorous and moderate activities (*P* > 0.05). Statistically, a significant difference was observed between high, low, and moderate and low groups for walking activities; however, the difference between high and moderate groups was statistically nonsignificant. All groups exhibited significant differences for total MET minutes per week (Additional file 1: Table S1).

**Table 3:** Physical activity and sitting time of participants following introduction of lockdown measures classified on the basis of IPAQ-SF between categories

Physical activity category	Vigorous (in min/week)	Moderate (in min/week)	Walking (in min/week)	Sitting (in min/week)	Total MET min/week
**Low** **(*n*-210. 102m; 108f)**	31.71 (100.7)	62.85 (76.23)	178.15 (121.3)	2729.6 (1407.7)	1309.82 (723.2)
**Moderate** **(*n*-99.** **57m; 42f)**	94.1 (103.7)	178.2 (317.6)	460.7 (517.3)	2470.4 (1312.5)	3292.63 (2717.8)
**High** **(*n*-63. 41m; 22f)**	185.5 (165.5)	292.4 (345.4)	610.8 (558.2)	1957.2 (1064.1)	4298.5 (3005.8)
**Total** **(*n*** **= 372; 200m, 172f)**	104.3 (122.6)	216 (373.4)	416 (398.6)	2965.3 (1261)	2772 (2867)

*MET* metabolic equivalents

Pearson product correlation coefficient analysis exhibited a weak inverse significant association between education (*r* = − 0.256, *P* < 0.05), marital status (*r* = − 0.242, *P* < 0.05), and those who scored the moderate level of physical activity on the IPAQ-SF (Table 4). A weak association was observed between the sitting activity and the SES level of participants (*r* = 0.098, *P* < 0.05), whereas no association was observed between other variables (*P* > 0.05). The results of regression analysis exhibited no statistical predictions between marital status, education, and moderate level of physical activity (*R*^2^ = 0.067; *P* > 0.05), and SES and sitting activity (*R*^2^ = 0.010; *P* > 0.05) (Additional file 1: Table S2).

**Table 4:** Correlation between demographic characteristics and physical activity categories among participants (*n* = 372) after lockdown

	Age	Sex	Education	Employment status	Marital status	SES
**Vigorous** **(in min/week)**	− 0.158 (*p* = 0.171)	− .106 (*p* = 0.361)	− .150 (*p* = 0.192)	− .174 (*p* = 0.129)	− .132 (*p* = 0.253)	− 0.04 (*p* = 0.720)
**Moderate** **(in min/week)**	− 0.140 (*p* = 0.227)	− 0.05 (*p* = 0.625)	− 0.256* (*p* = 0.026)	− 0.04 (*p* = 0.706)	− 0.242* (*p* = 0.035)	− 0.004 (*p* = 0.975)
**Walking** **(in min/week)**	− 0.202 (*p* = 0.708)	0.029 (*p* = 0.803)	− 0.162 (*p* = 0.160)	0.099 (*p* = 0.391)	0.029 (*p* = 0.805)	0.201 (*p* = 0.084)
**Sitting** **(in min/week)**	− 0.038 (*p* = 0.740)	0.108 (*p* = 0.349)	− 0.202 (*p* = 0.078)	− 0.063 (*p* = 0.585)	− 0.198 (*p* = 0.085)	0.098* (*p* = 0.041)
**Total MET/min**	− 0.233* (*p* = 0.042)	− 0.066 (*p* = 0.566)	− 0.223 (*p* = 0.051)	− 0.063 (*p* = 0.584)	− 0.111 (*p* = 0.338)	− 0.132 (*p* = 0.257)

*P < 0.05

## Discussion

The present study exhibited that the number of physical activities undertaken by a majority of the respondents was low and compromised by the pandemic. Participants who were free of COVID-19 symptoms and chronic diseases were recruited as these vulnerable groups would have taken precautionary measures and restricted their physical activity to protect themselves from the severity of COVID-19. The “stay-at-home” ordered by many governments worldwide, aiming at containing the spread of COVID-19, might have changed activity patterns. Reports from Italy and China have exhibited that COVID-19 lockdown was associated with a reduction in sports activities, increase in sleeping time and screen time [19], and difficulty for the public to continue standard physical activity patterns. A total of 138 people were engaged in recreational activities before lockdown. This figure was higher than the results (93.1%) reported in Tamil Nadu by Anjana et al. 2014 [5]. Another study exhibited that only 6.7% of the urban sample of Tamil Nadu engaged in recreational physical activity [20]. Other studies conducted in India have reported similar low engagement levels of the participants in leisure-time physical activities [6].

Several respondents (228/372; 61.29%) have reported a reduction in their physical activities during the lockdown phase, and only 86 (23.11%) of the respondents engaged in recreational physical activities during this period. Even those who committed (30/86; 34.88%) reported a reduction in the exercise intensity during the lockdown phase. Moderate-intensity physical exercises stimulated cellular immunity. On the other hand, moderate- to high-intensity physical activity of more than 90-min duration without adequate rest can reduce cellular immunity [21, 22]. Therefore, regular physical exercises should be encouraged as a preventive measure for health problems even during the quarantine period to fight against the pandemic. Numerous respondents reported engaging in recreational physical activities once the lockdown was lifted (151/372; 40.59%). However, the respondents reported low physical activity intensities even after lifted lockdown (216/372; 58.06%).

Although countries continue to report active COVID-19 cases, the ongoing health needs of the public must be attended to normalize the functioning of communities. Given the anticipated changes associated with pandemics, such as suspension of fitness centers, closure of public places and social isolation, the population’s physical activity status, and mental health get affected. Additionally, few weeks of physical inactivity can increase the risk of cardiovascular diseases [23] and metabolic maladaptation [8, 9], including a rise in inflammatory cytokines. Because no specific drug treatment exists for SARS-CoV-2 and not all people have received the COVID-19 vaccine, public health measures, and nonpharmacological treatment options have gained significance [23].

Low physical activities were reported by 210 (5.45%) participants, whereas 99 (26.61%) and 63 (16.93%) participants reported being engaged in moderate and high levels of physical activities. These reports are concurrent with the 71% inactivity reported in the urban residents of Tamil Nadu by Anjana et al. 2014 [5] and 63.3% inactivity reported in another study [20].

No difference in sitting time was observed among all 3 categories (high, moderate, and low-intensity activity categories). Physical inactivity and sedentary behavior [24, 25] are seen as long-term pandemics, leading to morbidity and mortality with the physical inactivity pattern expected to persist even after the COVID-19 pandemic [13]. Additionally, increased sitting time has been identified as an independent predictor of adverse health outcomes, [26] and every additional hour of sitting has been touted to increase annual healthcare costs in older adults [27]. A study by Du et al. 2019 [28] reported an increase in sitting time and no changes in physical activity patterns between 2007–2008 and 2015–2016 in the USA. Although the effect of pandemics on physical activities and sitting time is unknown, past experience has demonstrated that such pandemics can increase physical inactivity. Therefore, the increased physical inactivity and sitting activity during this pandemic may negatively impact the outcomes of future pandemics.

A previous work has reported that marital status, sex, education, employee category, and SES influenced the amount of time to engage in physical activity [29]. However, no significant association was observed between these parameters in the present study. This may be due to the timing of data collection when initiatives such as work from home and online learning are still a common practice. The sudden spike in the number of daily COVID-19-positive cases ensures that remote working and learning may be extended further, which may blur the role of family, work, and home commitments [30].

The results of the present study and periodical surveys monitoring the physical activities of the population may be used to watch the existing trends. The main limitation of the present study is that an online questionnaire and a “7-day recall" approach, which may not reflect the actual physical activities undertaken by the population, was used. Additionally, questions about physical activity patterns about a year ago may be subjected to recall bias. Information regarding other lifestyle factors such as smoking or drinking habits, sleep patterns, and diet were not collected. Furthermore, data collection was limited to the English-speaking population of a single city. Using social media platforms to recruit participants could limit the generalization of these data to other populations. The probability of variation in understanding the questions cannot be ruled out, too. Future studies using objective measurements may provide the basis for evaluating the long-term influence of reduced physical activity during pandemics. The role of social isolation and loneliness, distress and anger, job security, and loss of income due to COVID-19 as confounding factors could not be ruled out too. Despite these limitations, we recruited an adequate sample size reflecting the targeted population. The questions in the questionnaire easy enabled recall during the pandemic period and may be considered the actual lifestyle changes during that period. Further, the methods employed were the most feasible to reach a wider population. The data and results of this study may be used by policymakers to formulate guidelines for future lockdown and relaxation guidelines.

## Conclusion

The majority of the study population was physically inactive, and COVID-19 had both immediate and long-term impacts on self-reported physical activity patterns among the study sample. Clear guidelines regarding the intensity and type of physical activity patterns to be followed may assist the population in meeting the recommended physical activity levels.

## Supplementary Information

**Additional file 1: Supplementary file 1.** The questionnaire. **Supplementary Table 1.** Results of Tukey post-hoc test between categories classified on the basis of IPAQ-SF. **Supplementary Table 2.** Regression analysis of significant variables predicting physical activity categories among participants (n=372) after lockdown.

## Data Availability

The data that support the findings of this study are available from the corresponding author, [SG], upon reasonable request.

## Abbreviations

IPAQ: International physical activity questionnaire
IPAQ-SF: International Physical Activity Questionnaire-Short Form
SES: Socioeconomic status
MET: Metabolic equivalent of task
BMI: Body mass index
