# Supplementary material for: A cross-sectional study on the influence of COVID-19 pandemic on physical activity patterns among residents in a South Indian City
Source: Bull Fac Phys Ther. 2022 Aug 24;27(1):31. doi: 10.1186/s43161-022-00092-w (PMC9398494; doi:10.1186/s43161-022-00092-w)
Supplement: Supplementary file 1 — Additional file 1: Supplementary file 1. The questionnaire. Supplementary Table 1. Results of Tukey post-hoc test between categories classified on the basis of IPAQ-SF. Supplementary Table 2. Regression analysis of significant variables predicting physical activity categories among participants (n=372) after lockdown. [file 43161_2022_92_MOESM1_ESM.docx]

Supplementary File

**The questionnaire**

1. Age: years

2. Gender：

□Male

□Female

3. Height： cm

4. Weight:___ kg

5. What is your education level?

□Graduate

□Post-Graduate

□ PhD or above

6. Are you currently employed?

□ Yes

□ No

7. If yes, are you

□ Working from home

□ Working from office / outdoor jobs.

7. What is the total monthly household income of your family? (in INR)

□<12,000

□ 12,000-19,999

□ 20,000-59,999

□ 60,000-99,999

□ 100,000-199,999

□≥200,000

8. What is your marital status?

□ Single

□ Married

□ Widowed / Separated

9. Who else is CURRENTLY living in your household? Please tick all that apply

□live alone

□ living with my partner

□ living with partner and kids

□ living with other family members

□ living with colleagues/ room mates

9. In general, how would you rate your health?

- - Excellent
  - Very good
  - Good
  - Fair
  - Poor

10. Do you suffer from any chronic diseases?

- Arthritis
- Asthma
- Cancer
- Cystic fibrosis
- Chronic obstructive pulmonary disease (COPD)
- Diabetes
- Epilepsy
- Heart disease
- High blood pressure
- High cholesterol
- HIV/ AIDS
- Mental health condition
- Multiple Sclerosis
- I have had an organ transplant
- I have a condition that makes me much more likely to get infections
- I am taking medicine that weakens my immune system
- Dementia, Parkinson’s or other neurological disease
- Prefer not to say
- None of these

Please specify duration …….( in months)

11. In general, how would you rate your daily chores before lockdown?

- Light
- Moderate
- Vigorous

12. Did you engage yourself in leisure time physical activity before lockdown?

Yes/ No

If yes, please specify duration …………

13. How would you rate your leisure time activity before lockdown?

- Light
- Moderate
- Vigorous

14. How many days a week did they engage in leisure time physical activities?

- - All days
  - Minimum 5 days a week
  - Minimum 3 days a week
  - Less than 3 days

15. How would the respondent rate their daily physical activities during lockdown phase compared to their daily physical activities before lockdown?

- Same as previous intensities
- less than previous intensities
- more than previous intensities

16.Did the respondent engage in leisure time physical activities during lockdown phase?

Yes/ No

17. If yes, how would they rate their leisure time physical activities compared to their daily leisure time physical activities before lockdown?

- Same as previous intensities
- less than previous intensities
- more than previous intensities

18.How would the respondent rate their daily physical activities during unlockdown phase compared to their daily physical activities before lockdown?

- Same as previous intensities
- less than previous intensities
- more than previous intensities

19. .Did the respondent engage in leisure time physical activities during unlockdown phase?

Yes/ No

20. If yes, how would they rate their leisure time physical activities compared to their daily leisure time physical activities before lockdown?

- Newly started
- Same as previous intensities
- less than previous intensities
- more than previous intensities

21. Did you/ any members of your household suffer/ suffering from any of the symptoms of Covid-19? ( fever, cough, shortness of breath, loss of sense of smell , loss of sense of taste ,decrease in appetite (skipping meals) ,diarrhoea ,nauseas and/or vomiting, abdominal pain/tummy ache, chills (feeling too cold), difficulty sleeping, felt more tired than normal, severe fatigue, sneezing, chest pain / tightness, sore throat, hoarse voice) (Please do NOT include symptoms you experience on a regular basis due to a health condition you already know about)

Yes/No

If yes, mention the symptom…., and duration….

21.Was the respondent/ any members of his household affected with covid-19?

Yes/No

22. If yes, was the respondent

- quarantined at home
- Self-isolated
- required hospital admission

23. Did the respondent/ any members of their household shield himself/ herself as per the government guidelines for vulnerable groups?

□ Yes

□ No

**Supplementary Table 1**

**Results of Tukey post-hoc test between categories classified on the basis of IPAQ-SF**

| **Categories as per IPAQ-SF** | **Groups High and low** | | | **Groups High and moderate** | | | **Groups moderate and low** | | |
| --- | --- | --- | --- | --- | --- | --- | --- | --- | --- |
|  | **Mean diff** | **95% C.I** | **p** | **Mean diff** | **95% C.I** | **p** | **Mean diff** | **95% C.I** | **p** |
| **Vigorous**  (in min/week) | 52.19 | (32.71,181.68) | <0.05* | 33.16 | (28.27,78.06) | <0.05* | 24.03 | (6.07,31.73) | >0.05 |
| **Moderate**  (in min/week) | 62.73 | (14.03,107.42) | <0.05* | 36.59 | (12.77,68.40) | <0.05* | 38.13 | (16.67,42.49) | >0.05 |
| **Walking**  (in min/week) | 146.46 | (58.57,81.05) | <0.05* | 50.09 | (38.37,61.82) | <0.05* | 93.71 | (44.13,95.29) | <0.05* |
| **Total MET**(in min/week) | 4188.67 | (3514.24,4863.09) | <0.05* | 3005.86 | (2302.22,3709.50) | <0.05* | 1182.80 | (848.02,1517.58) | <0.05* |

**Supplementary Table 2**

**Regression analysis of significant variables predicting physical activity categories among participants (n=372) after lockdown.**

| **VARIABLE** | **B** | **SE B** | **B** | **P value** |
| --- | --- | --- | --- | --- |
| **MODERATE PHYSICAL ACTIVITY** | | | | |
| **CONSTANT** | 3.39 | 1.01 |  | 0.01 |
| **EDUCATION** | -0.90 | 1.10 | -0.186 | 0.417 |
| **MARITAL STATUS** | -0.34 | 0.97 | -0.80 | 0.727 |
| **SITTING ACTIVITY** | | | | |
| **CONSTANT** | 5.171 | 2.032 |  | 0.013 |
| **SES** | 0.710 | 0.841 | 0.098 | 0.401 |
